# A Novel Ensemble-Based Machine Learning Algorithm to Predict the Conversion From Mild Cognitive Impairment to Alzheimer's Disease Using Socio-Demographic Characteristics, Clinical Information, and Neuropsychological Measures

**DOI:** 10.3389/fneur.2019.00756

**Published:** 2019-07-16

**Authors:** Massimiliano Grassi, Nadine Rouleaux, Daniela Caldirola, David Loewenstein, Koen Schruers, Giampaolo Perna, Michel Dumontier, Alzheimer's Disease Neuroimaging Initiative

**Affiliations:** ^1^Department of Clinical Neurosciences, Hermanas Hospitalarias, Villa San Benedetto Menni Hospital, FoRiPsi, Albese con Cassano, Italy; ^2^Department of Biomedical Sciences, Humanitas University, Milan, Italy; ^3^Faculty of Science and Engineering, Institute of Data Science, Maastricht University, Maastricht, Netherlands; ^4^Department of Psychiatry and Behavioral Sciences, Miller School of Medicine, University of Miami, Miami, FL, United States; ^5^Wien Center for Alzheimer's Disease and Memory Disorders, Mount Sinai Medical Center Miami Beach, Miami Beach, FL, United States; ^6^Center for Cognitive Neuroscience and Aging, Miller School of Medicine, University of Miami, Miami, FL, United States; ^7^Research Institute of Mental Health and Neuroscience and Department of Psychiatry and Neuropsychology, Faculty of Health, Medicine and Life Sciences, Maastricht University, Maastricht, Netherlands

**Keywords:** Alzheimer's disease, clinical prediction rule, machine learning, mild cognitive impairment, personalized medicine, precision medicine, neuropsychological tests

## Abstract

**Background:** Despite the increasing availability in brain health related data, clinically translatable methods to predict the conversion from Mild Cognitive Impairment (MCI) to Alzheimer's disease (AD) are still lacking. Although MCI typically precedes AD, only a fraction of 20–40% of MCI individuals will progress to dementia within 3 years following the initial diagnosis. As currently available and emerging therapies likely have the greatest impact when provided at the earliest disease stage, the prompt identification of subjects at high risk for conversion to AD is of great importance in the fight against this disease. In this work, we propose a highly predictive machine learning algorithm, based only on non-invasively and easily in-the-clinic collectable predictors, to identify MCI subjects at risk for conversion to AD.

**Methods:** The algorithm was developed using the open dataset from the Alzheimer's Disease Neuroimaging Initiative (ADNI), employing a sample of 550 MCI subjects whose diagnostic follow-up is available for at least 3 years after the baseline assessment. A restricted set of information regarding sociodemographic and clinical characteristics, neuropsychological test scores was used as predictors and several different supervised machine learning algorithms were developed and ensembled in final algorithm. A site-independent stratified train/test split protocol was used to provide an estimate of the generalized performance of the algorithm.

**Results:** The final algorithm demonstrated an AUROC of 0.88, sensitivity of 77.7%, and a specificity of 79.9% on excluded test data. The specificity of the algorithm was 40.2% for 100% sensitivity.

**Conclusions:** The algorithm we developed achieved sound and high prognostic performance to predict AD conversion using easily clinically derived information that makes the algorithm easy to be translated into practice. This indicates beneficial application to improve recruitment in clinical trials and to more selectively prescribe new and newly emerging early interventions to high AD risk patients.

## Introduction

Alzheimer's Disease (AD) is a neurodegenerative disease characterized by progressive memory loss, cognitive impairment and general disability; AD is the most common cause of dementia of the Alzheimer's type. The progression of AD comprises a long, unnoticed preclinical stage, followed by a prodromal stage of Mild Cognitive Impairment (MCI) that leads to severe dementia and eventually death (1). While no disease-modifying treatment is currently available for AD, a large number of drugs are in development and encouraging early-stage results from clinical trials provide for the first time a concrete hope that one or more therapies may become available in a few years (2). As the progression of the neuropathology in AD starts years in advance before clinical symptoms of the disease become apparent and progressive neurodegeneration has irreversibly damaged the brain, emerging treatments will likely have the greatest effect when provided at the earliest disease stages. Thus, the prompt identification of subjects at high risk for conversion to AD is of great importance.

The ability to identify declining individuals at the prodromal AD stage provides a critical time window for early clinical management, treatment & care planning and design of clinical drug trials (3). Precise identification and early treatment of at risk subjects would stand to improve outcomes of clinical trials and reduce healthcare costs in clinical practice. However, simulations also suggest that the health care system is not prepared to handle the potentially high volume of patients who would be eligible for treatment (2).

MCI represents (currently) the earliest clinically detectable stage of a potential ongoing progression toward AD or other dementias. The cognitive decline in MCI is abnormal given an individual's age and education level, but does not interfere with daily activities, and thus does not meet criteria for AD. However, only 20–40% of individuals will progress to AD within 3 years, with a lower rate of conversion reported in epidemiologic samples than in clinical ones (4, 5).

Currently, there are no means to provide patients diagnosed with MCI with an early prognosis for conversion to AD. While changes in several biomarkers prior to developing AD have been reported, no single biomarker appears to adequately predict the conversion from MCI to AD with an acceptable level of accuracy. As such, there is increasing evidence that the use of a combination of biomarkers can best predict the conversion to AD (3, 6–9).

In the current age of big data and artificial intelligence technologies, considerable effort has been dedicated in developing machine learning algorithms that can predict the conversion to AD in subjects with MCI. In almost all medical fields, the introduction into research and clinical practice of machine learning based decision-making tools, and more in general the shift toward a personalized medicine paradigm, is currently a debated topic and viewed as an opportunity to improve clinical outcomes. Such objective tools may provide individual predictions with a certain degree of confidence based on information that can be collected about the subject, so that researchers and clinicians may be supported by these predictions in order to take better and more effective decisions (10).

So far, many studies focused on predicting the conversion of AD in MCI patients using different combinations of data including brain imaging, CSF biomarkers, genotyping, demographic and clinical information, and cognitive performance, achieving varying levels of accuracy [(7, 11–19); see (20, 21)] for a recent review of the most performing algorithms presented in the scientific literature so far). However, while combining different biomarkers improves model accuracy, there is a lack of consistency regarding a specific combined AD prediction model and translation into practice is still lacking. One possible reason for this is that current algorithms generally rely on expensive and/or invasive predictors, such as brain imaging or CSF biomarkers. As such, these studies only serve the purpose of a proof-of-concept, without being further tested in independent and clinical samples.

The current study aimed to develop a clinically translatable machine learning algorithm to predict the conversion to AD in subjects with MCI within a 3-year period, based on fast, easy, and cost-effective predictors. Specifically, we chose to develop a variety of machine learning algorithms based on distinct supervised machine learning techniques and subsets of the considered predictors, followed by a weighted average rank ensemble strategy on the predictions provided by the various algorithms to obtain a final, more accurate prediction. Our hypothesis was that high predictive accuracy could be obtained using the above-mentioned approach with simple and non-invasive predictors. We used data obtained from the Alzheimer's Disease Neuroimaging Initiative (http://adni.loni.usc.edu/) with a particular consideration for socio-demographic and clinical information, and neuropsychological test scores rather than using complex, invasive, and expensive imaging or CSF predictors.

## Materials and Methods

### ADNI

Data used in the preparation of this article were obtained from the Alzheimer's Disease Neuroimaging Initiative (ADNI) database (adni.loni.usc.edu). The ADNI was launched in 2003 as a public-private partnership, led by Principal Investigator Michael W. Weiner, MD. The primary goal of ADNI has been to test whether serial magnetic resonance imaging (MRI), positron emission tomography (PET), other biological markers, and clinical and neuropsychological assessment can be combined to measure the progression of MCI and early AD. It contains data of a large number of cognitive normal, MCI, and AD subjects recruited in over 50 different centers in US and Canada with follow-up assessments performed every 6 months.

For this study, we used a subset of the ADNI dataset called ADNIMERGE that includes a reduced selection of more commonly used variables (i.e., demographic, clinical exam total scores, MRI and PET variables). This subset is part of the official dataset provided by ADNI.

### Subjects

Data regarding 550 subjects with MCI and with available diagnostic follow-up assessments for at least 3 years were included in the study. The most relevant inclusion criteria of ADNI studies are the following: age between 55 and 90; six grade education or work history; subjects had to be fluent English/Spanish speakers; Geriatric Depression Scale score <6; good general health; no use of excluded medications (e.g., medications with anticholinergic properties) and stability for at least 4 weeks of other allowed medications; Hachinski ischemic score scale ≤ 4. A complete description of the ADNI study inclusion/exclusion criteria, including the full list of excluded and permitted medications, can be found in the ADNI General Procedure Manual, pages 20–25 (link: https://adni.loni.usc.edu/wp-content/uploads/2010/09/ADNI_GeneralProceduresManual.pdf).

The diagnosis of MCI was performed with the following criteria: memory complaint by subject or study partner that is verified by a study partner; abnormal memory function documented by scoring below the education adjusted cutoff on the Logical Memory II subscale (Delayed Paragraph Recall) from the Wechsler Memory Scale—Revised, which is ≤ 11 for 16 or more years of education, ≤ 9 for 8–15 years of education, and ≤ 6 for 0–7 years of education; Mini-Mental State Exam (MMSE) score between 24 and 30; Clinical Dementia Rating (CDR) score of 0.5; Memory Box score at least of 0.5; general cognition and functional performance sufficiently preserved such that a diagnosis of AD cannot be made.

Subjects were classified as converters to probable AD (cAD; *n* = 197, 35.82%) if they satisfied the National Institute of Neurological and Communicative Disorders and Stroke/Alzheimer's Disease and Related Disorders Association criteria for AD [28] during at least one of the follow-up assessments occurred within 3 years from the baseline investigation, as well as having a MMSE score between 20 and 2. Otherwise, they were classified as non-converters to AD (NC; *n* = 353, 64.18%).

The study procedures were approved by the institutional review boards of all participating centers to the Alzheimer's Disease Neuroimaging Initiative, and written informed consent was obtained from all participants or their authorized representatives.

### Feature Extraction

Considering our aim to employ only predictors that are either already routinely assessed or easily introducible in clinical practice, and that are not perceived as invasive by patients, we decided to take into account only variables in the ADNIMERGE dataset that regards diagnostic subtypes, sociodemographic characteristics, clinical and neuropsychological test scores. Some of these variables were not available for all recruited subjects and it was a priori decided to remove variables with >20% missing values. Only the Digit Span Test score (DIGIT) exceeded the cut-off (52.73%) and was not used in our analysis. The following variables were used:

- Sociodemographic characteristics: sex, age (in years), years of education, and marital status (never married, married, divorced, widowed, unknown).
- Subtypes of MCI: Early or Late MCI according to their score in the Logic Memory subscale of the Wechsler Memory Scale—Revised (22), adjusted for the years of education. 9–11 Early MCI and ≤ 8 Late MCI for 16 or more years of education; 5–9 Early MCI and ≤ 4 Late MCI for 8–15 years of education; 3–6 Early MCI and ≤ 2 Late MCI for 0–7 years of education.
- Clinical scales: CDR (23) was used to characterize six domains of cognitive and functional performance in AD and related dementias: Memory, Orientation, Judgment & Problem Solving, Community Affairs, Home & Hobbies, and Personal Care. The rating is obtained through a semi-structured interview of the patient together with other informants (e.g., family members). Sum of Boxes score was used in the current analyses (CDRSB). The score of the Functional Assessment Questionnaire (FAQ) (24), an a informant-based clinician-administered questionnaire which assess the functional daily-living impairment in dementia, was also used in the analyses.
- Neuropsychological tests: MMSE (25) is a 30-point questionnaire that is used measuring cognitive impairment. All MCI subjects has a score of 24 of more at baseline. The Cognitive Subscale Alzheimer's Disease Assessment Scale (ADAS) (26) is made of 11 tasks that include both subject-completed tests and observer-based assessments, assessing the memory, language, and praxis domains. The result is a global final score ranging from 0 to 70, based on the sum of the scores of the single tasks (ADAS11). Beyond the ADAS11 score, the ADNI study included also an additional test of delayed word recall and a number cancellation or maze task, which are further summed to have a new total score that ranges from 0 to 85 (ADAS13). In addition, the score of the task 4 (Word Recognition, ADASQ4) was included in the ADNIMERGE dataset. All these three ADAS scores were initially considered as predictors in the analyses. The Rey Auditory Verbal Learning Test (RAVLT) (27) is a cognitive test used to evaluate verbal learning and memory. All the immediate (RAVLT-I), learning (RAVLT-L), forgetting (RAVLT-F), and percent forgetting (RAVLT-PF) scores were included in the ADNIMERGE dataset and used in the analyses. Moreover, the total delayed recall score of the Logic Memory subtest of the of the Wechsler Memory Scale-Revised (28) (LDT), which assess verbal memory, and the time to complete of the Trial Making Test version B (TMTBT) (29), which assess visual-motor coordination and attentive functions. A summary of the abbreviations of all neuropsychological tests can be found in Table 1.

**Table 1:** Abbreviations of neuropsychological tests.

ADAS11	Cognitive Subscale (11 items) Alzheimer's Disease Assessment Scale
ADAS13	Cognitive Subscale (13 items) Alzheimer's Disease Assessment Scale
ADASQ4	Task 4 of the Cognitive Subscale (11 items) Alzheimer's Disease Assessment Scale
CDRSB	Sum of Boxes score of the Clinical Dementia Rating Scale
DIGIT	Digit Span Test score
FAQ	Functional Activities Questionnaire
LDT	Logic Memory subtest of the of the Wechsler Memory Scale-Revised
RAVLT	Rey Auditory Verbal Learning Test
RAVLT-F	Forgetting score of the Rey Auditory Verbal Learning Test
RAVLT-I	Immediate score of the Rey Auditory Verbal Learning Test
RAVLT-L	Learning score of the Rey Auditory Verbal Learning Test
RAVLT-PF	Percent forgetting score of the Rey Auditory Verbal Learning Test
TMTBT	Trial Making Test, version B


Taken together, 14 continuous, 2 dichotomous and 1 polytomous categorical features were initially considered. The full list is available in Table 2.

**Table 2:** Descriptive statistics.

**Continuous predictors**		**Non-converters**		**Converters**		**Missing values**	
		**Mean**	**S.D**.	**Mean**	**S.D**.	***N***	**%**
Age		72.42	7.54	74.19	6.88	/	/
Years of education		16.18	2.74	15.74	2.83	/	/
CDRSB		1.26	0.70	1.95	1.01	/	/
ADAS11		8.67	3.78	12.94	4.26	1	0.18
ADAS13		13.89	5.81	21.05	5.72	3	0.55
ADASQ4		4.61	2.35	7.16	2.04	/	/
MMSE		28.01	1.71	26.85	1.72	/	/
RAVLT-I		37.84	10.47	28.05	6.74	/	/
RAVLT-L		4.76	2.59	2.90	2.11	/	/
RAVLT-F		4.37	2.46	5.20	2.30	/	/
RAVLT-PF		51.09	30.92	78.20	28.04	/	/
LDT		6.84	3.12	3.59	2.89	/	/
DIGIT		40.24	10.42	34.86	11.02	290	52.73
TMTBT		100.30	49.56	141.24	79.66	4	0.73
FAQ		1.76	2.75	5.81	5.00	4	0.73
**Categorical predictors**		**Non-converters**		**Converters**		**Missing values**	
		***N***	**%**	***N***	**%**	***N***	**%**
Sex	Male	220	62.32	118	59.90	/	/
	Female	133	37.68	79	40.10		
Subtype of MCI	Early	196	47.88	22	11.17	/	/
	Late	184	52.12	175	88.83		
Marital status	Never married	6	1.70	3	1.52	3	0.55
	Married	267	75.64	161	81.73		
	Divorced	35	9.92	13	6.60		
	Widowed	42	11.90	20	10.15		

*S.D, Standard Deviation; N, numbers of subjects*.

### Dataset Division in 5 Site-Independent, Stratified Test Subsets

The entire dataset was divided in five mutually exclusive data subsets. These five subsets were created in order to satisfy the following criteria: every subset has to include roughly 20% of the cases; all subjects from each of the 58 different recruitment sites has to be allocated into the same subset; every subset has to include roughly the same percentage of cAD as observed in the entire dataset (35.82%). In order to accomplish a division in 5 folds which satisfies all these criteria, 10,000 different subsets were generated by progressively adding all subjects from a randomly chosen recruiting site, until the included cases ranged between 19 and 21% of the entire sample. Then, only those subsets whose percentage of cAD ranged between 35.52 and 36.12% were retained, which was satisfied in 567 (5,67%) out of the generated subsets. Finally, all possible combinations of five of the retained subsets were created in order to identify whether in any of these combinations covered the entire dataset without any repetition of cases. The entire process took around 4 h of computation (on a Linux server with 2.20GHz Intel Xeon E5-2650 v4 CPUs), and successfully found a single combination of five subsets that satisfied all the desired criteria (Table 3).

**Table 3:** Allocation of the ADNI study recruitment sites in the five subsets.

**Subset**	**Recruiting sites**																		**Non-converters**		**Converters**	
																			***N***	**%**	***N***	**%**
A	6	12	18	21	126	127	128	137											74	64.35	41	35.65
B	2	3	9	23	24	29	36	37	94	99	114								70	64.22	39	35.78
C	7	13	14	33	41	67	73	98	100	109	116								70	64.22	39	35.78
D	5	16	22	27	31	35	123	130	141	153									70	64.22	39	35.78
E	10	11	19	32	51	52	53	57	62	68	72	82	129	131	133	135	136	941	69	63.89	39	36.11

*Code of the recruitment sites are those available in the ADNIMERGE file, following the coding convention used in the ADNI study*.

All the missing value imputation, feature transformation and selection procedures, model training with cross-validation, and ensembling of different algorithms predictions described in the following paragraphs were performed in five distinct repetitions (named A-E) of the analyses, each time using the cases included in four of the five subsets and blindly to the remaining subset that were used as a test subset. The same missing value imputation, feature transformation and selection applied during training in the other four subsets were applied to the test subset. The predictive algorithms and their ensembling procedure developed in the other 4 subsets were tested against the test subset to obtain an estimate of the generalized performance in an independent sample of cases recruited in sites different from the ones used for training^1^.

### Feature Transformation and Selection

Imputation was performed for variables with missing values using the median for continuous features and using the mode for categorical features. Continuous variables were standardized (mean = 0, standard deviation = 1) and non-dichotomous categorical variables were dichotomized using one-hot encoding, i.e., re-coding them in a new dichotomous variable for each class of the categorical variable, with 1 indicating the occurrence of that class and 0 the occurrence of any other class of the variable.

In case groups of variables resulted highly correlated (pairwise *r* > = 0.75), principal component analysis was used to calculate principal components and the original variables were substituted with all the components with eigenvalues > = 1.

All features were initially used during training (feature set 1). Moreover, three feature subsets were additionally created based on different selection strategies in order to include only those that are the most informative. A filtering procedure was applied to create reduced sets of features based on their bivariate statistical association (*p* < 0.05) with the outcome using independent sample *t*-test for continuous predictors and Fisher's exact test for both dichotomous and one-hot encoded polytomous features (feature subset 2). Two cross-validated recursive feature elimination procedures (also known as “wrapper” procedures) with Logistic Regression (LR, feature subset 3) and Random Forest (RF, feature subset 4) (30) were also applied. In particular, the latter strategy was chosen because it has previously proved to be efficacious in selecting a relevant feature subset (20).

### Machine Learning Techniques

Several machine learning procedures that can be used to solve classification problems exists. We used 13 supervised techniques: LR, Naive Bayes (NB) (31), L1 and L2 regularized logistic regression or Elastic Net (EN) (32), Support Vector Machine (33) with linear (SVM-Linear), radial basis function (SVM-RBF), and polynomial (SVM-Poly) kernels with Platt scaling (34), k-Nearest Neighbors algorithm (kNN) (35), Multi-Layer Perceptrons with either one or two hidden layers and trained with either a full-batch gradient descent or adam (36) algorithms (MLP1-Batch, MLP2-Batch, MLP1-Adam, MLP2-Adam), RF, and Gradient Tree Boosting of Decision Trees (GTB) (37). All analyses were parallelized on a Linux server equipped with four 12-core Intel Xeon CPU E5-2650 v4 @ 2.20GHz and were performed in Python 3.6 (38), using the implementation of the machine learning techniques available in the Scikit-Learn library (39).

### Hyper-Parameter Optimization

Machine learning techniques usually have one or more hyper-parameters that allow a different tuning of the algorithm during the training process. Different values of these hyper-parameters lead to algorithms with different predictive performances with the goal of obtaining the best possible performance when applied to cases that are not part of the training set. In order to optimize such hyper-parameters for each ML techniques used in this study, each model was trained with 50 random hyper-parameter configurations, and 50 further configurations were progressively estimated with a Bayesian optimization approach. Instead of a random generation, Bayesian optimization aims to estimate which is the hyper-parameter configuration that would maximize the performance of the algorithm starting from the previously attempted ones, based on the assumption that it exists a relationship between the various hyper-parameter values and the performance achieved by the algorithm. Bayesian optimization is expected of being able to identify better hyper-parameter configurations, and in a reduced number of attempts, than just trying to generate them at random. Estimation was performed with Gaussian Processes, as implemented in the Scikit-Optimized library (https://scikit-optimize.github.io/).

The Area Under the Receiving Operating Curve (AUROC) was used as performance metric to be maximized. All the ML algorithms developed in this study output a continuous prediction score (range: 0–1; the closer to 1 the higher the predicted risk of conversion for that subject) and the AUROC value can be interpreted as the probability that a randomly selected cAD subject will receive a higher output score than a randomly selected NC subject. The AUROC value is 0.5 when the algorithm makes random predictions and 1 in case it is always correct in making predictions. AUROC is not affected by class imbalance and it is independent with respect to any specific threshold that is applied to perform a dichotomous prediction.

### Cross-Validation Procedure

The aim is to develop an algorithm that can achieve the best possible generalized performance and not to perform well only with the cases used in the training process. Cross-validation provides an estimate of such generalized performance for every hyper-parameter configuration. In cross-validation, the train sample is divided in several folds of cases that are held-out from the training process, with training iteratively performed with the remaining cases. After the training, the algorithm is finally applied on the held-out cases.

We applied the commonly used 10-fold cross-validation procedure, repeated 10 times to obtain a stable performance estimate. The fold creation was performed at random, stratifying (i.e. balancing) for the percentage of converters and non-converters in each fold. Finally, the 100 performance estimates of the algorithm available for each hyper-parameter configuration were averaged to provide a final point estimate of the generalized performance. The hyper-parameter configuration for each machine learning technique that demonstrated the best average cross-validated AUROC was retained.

### Weighted Rank Average of Single Algorithm Predictions

Using a collection of algorithms and combining their predictions instead of considering only the prediction coming from a single algorithm generally improves the overall predictive performance (40). This procedure is called ensembling and it is also the principles on which some individual techniques such as Random Forest and Gradient Boosting techniques are based.

Several different ensemble methods exist, which usually require a further independent data subset from both the training and test ones. This additional subset would be used to train how to optimally combine the various predictions generated by the single algorithms. Given the limited amount of data available in the current study, further reducing the size of the train sample may have undermined the predictive performance of the developed algorithms. Thus, we decided to apply a simple form of ensembling based on a weighted average of the rank predictions generated by all individual algorithms. This strategy is usually considered effective even though it does not require to develop any further machine learning meta-algorithm and to optimize its hyperparameters (41).

First, the ranks of the cross-validated continuous prediction scores of the train subset cases were calculated for each of the 52 developed algorithms, and rescaled in order to range between 0 and 1. Then, the arithmetic average of the rescaled ranks weighted for the cross-validated AUROC was calculated for each train subset case, representing the new continuous prediction scores for the train subset cases.

To generate the final continuous prediction scores of the test subset cases, at first 52 prediction scores for each test case were generated using all the 52 used algorithms. Then, the prediction score of each algorithm was substituted with the rescaled rank of the closest cross-validated train subset prediction score of that algorithm. Finally, the average of the rescaled ranks weighted for the cross-validated AUROC was calculated. This represents the final continuous prediction scores of each test subset cases.

### Testing Performance

The final continuous prediction scores of the five test subsets, which were obtained using the weighted rank average, were pooled and used to calculate the whole sample test AUROC. This represents the final estimate of the generalized site-independent AUROC that the algorithm is expected to achieve when it is applied to new cases. The 95% confidence interval (CI) of the AUROC was calculated with a stratified bootstrap procedure, with 10,000 resamples and applying the bias-corrected and accelerated (BCa) approach (42).

Different categorical cAD/NC predictions were generated for each case applying various thresholds to the final continuous prediction scores (i.e., a score equal or above the threshold indicated a cAD, otherwise a NC). First, the threshold values that maximized the balanced accuracy (i.e., the average between sensitivity and specificity) of the cross-validated train subsample ensemble predictions in each of the five analyses replication was identified and averaged in order to have a final unique threshold that was applied to the final continuous prediction scores. Moreover, the threshold values that generated sensitivity of 100, 97.5, 95, 90, 85, 80, 75% of the cross-validated train subsample ensemble predictions in each of the five analyses replication was identified, averaged and applied to the final continuous prediction scores.

Specificity (i.e., recall), sensitivity, positive predictive value (i.e., precision), negative predictive value, balanced accuracy and F1 score (i.e., the harmonic average of the sensitivity and positive predictive value) were calculated considering the pooled categorical predictions generated with the abovementioned thresholds, which represent the estimates of the generalized site-independent performance of the algorithm when applied to perform categorical predictions of cAD/NC in new cases, such that either the balanced-accuracy is aimed to be maximized or defined levels of sensitivity are aimed to be obtained.

### Feature Importance

To provide a general ranking of the importance of the predictors used in this study, we applied the same five train/test split protocol to iteratively develop logistic regression models using only a single feature, in the train subsets, and these models were applied to generate the continuous prediction scores in the five test subsamples. The scores of the test subsamples were finally pooled together and used to calculate the whole sample test AUROC for each predictor. This gives a metric of importance for each predictor that is independent from both the machine learning technique used and all other predictors inserted in the algorithm. The 95% confidence interval (CI) of also these AUROCs was calculated with a stratified bootstrap procedure, with 10,000 resamples and applying the bias-corrected and accelerated (BCa) approach (42).

## Results

Descriptive statistics of each feature in the cAD and NC groups are reported in Table 2. Statistics of continuous features are reported before the standardization was applied.

### Feature Transformation and Selection

Two groups of features correlated above the 0.75 threshold were identified, respectively the three ADAS scores (ADAS11, ADAS13, ADASQ4) and two of the RAVLT scores (RAVLT-F, RAVLT-PF). Such evidence equally resulted in all of the five training subsets. In all of the 5 subsets, only the first principal component of each group had an eigenvalue > = 1, and these were used to substitute the correlated features as predictors (ADAS-PC1, RAVLT-F-PC1).

Across the five training subsamples used in the analyses, each feature selection procedure selected only partially overlapping subsets of relevant features, as reported in Table 4. Thus, the feature sets 2, 3, and 4 used in the analyses were in part different across the training subsamples used in the five repetitions of the analyses. This evidence further justifies our choice of creating several site-independent train and test subsamples instead of just a single training and test split, in order to provide a better and more stable estimate of the generalized performance of the algorithm.

**Table 4:** Feature sets 2, 3, and 4 in each of the five replications of the analyses.

**Predictors**	**Feature set 2**					**Feature set 3**					**Feature set 4**				
	**A**	**B**	**C**	**D**	**E**	**A**	**B**	**C**	**D**	**E**	**A**	**B**	**C**	**D**	**E**
Age	x	x		x	x						x	x	x	x	x
Years of education											x	x	x	x	
CDRSB	x	x	x	x	x	x	x	x	x	x	x	x	x	x	x
ADAS-PC1	x	x	x	x	x	x	x	x	x	x	x	x	x	x	x
MMSE	x	x	x	x	x	x			x		x	x	x	x	
RAVLT-I	x	x	x	x	x	x	x	x	x	x	x	x	x	x	x
RAVLT-L	x	x	x	x	x	x			x		x	x	x	x	x
RAVLT-F-PC1	x	x	x	x	x	x	x	x	x	x	x	x	x	x	x
LDT	x	x	x	x	x		x		x	x	x	x	x	x	x
TMTBT	x	x	x	x	x	x	x	x	x	x	x	x	x	x	x
FAQ	x	x	x	x	x	x	x	x	x	x	x	x	x	x	x
Sex						x	x	x	x	x	x	x	x	x	
Subtype of MCI	x	x	x	x	x	x	x	x	x	x	x	x	x	x	
Marital status—Never married						x	x		x	x	x				
Marital status—Married						x	x	x	x	x	x	x			
Marital status—Divorced						x	x	x	x	x	x	x			
Marital status—Widowed						x	x	x	x	x	x	x			

*A-E indicates the 5 independent subsets in which the analyses have been replicated*.

Among the features, CDRSB, ADAS-PC1, RAVLT-I, RAVLT-F-PC1, TMTBT, and FAQ, were selected by all the three feature selection strategies in all of the five repetitions of the analyses, the subtype of MCI was discarded only once, LDT twice, RAVLT-L three times and MMSE four times. All the sociodemographic characteristics were all discarded at least 6 up to 11 times out of the 15 feature sets identified in the analyses.

### Performance of the Predictive Algorithm

The cross-validated AUROC results for each of the 52 models developed in each repetitions are reported in Table S1, which ranged from a minimum value of 0.83 to a maximum value of 0.90 for the models developed with feature set 1, from 0.84 to 0.90 for the models developed with feature set 2, from 0.84 to 0.89 for the models developed with feature set 3, and from 0.83 to 0.90 for the models developed with feature set 4. These results indicate a narrow difference of performance among different feature sets, as well as among different replications and techniques, which included simple linear models such LR and NB as well as ensembling technique such as RF and GBM. The cross-validated AUROC of the weighted rank average ensembling strategy in each fold is also reported in Table S1, which ranged from a minimum of 0.86 to a maximum of 0.89.

**Figure 1:**
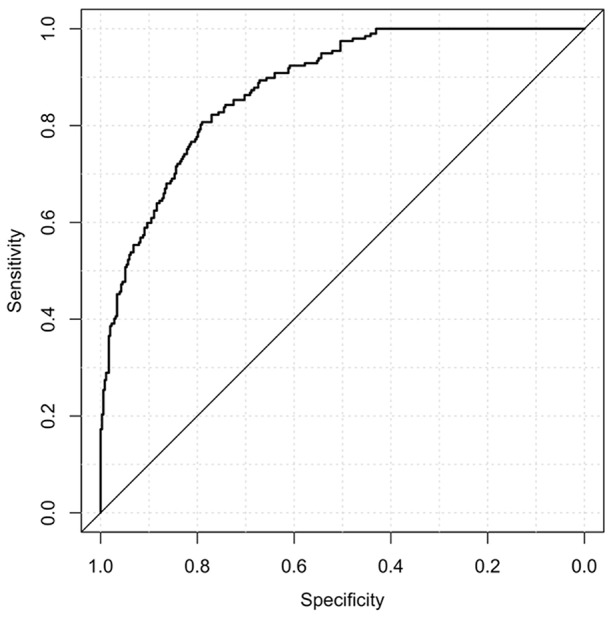
Area under the receiving operating curve of the pooled test predictions.

When the test continuous prediction scores obtained with the ensembling approach were pooled, the whole sample test AUROC resulted 0.88 (95% bootstrap CI 0.85–0.91), which is plotted in Figure 1.

Considering the categorical predictions generated with the threshold that maximized the training balanced accuracy, results indicated a sensitivity/recall of 77.7%, a specificity of 79.9%, a positive predictive value/precision of 68.3%, a negative predictive value of 86.5%, a balanced accuracy of 0.79, and F1-score of 0.73. Results generated applying the other thresholds are reported in Table 5.

**Table 5:** Test performance of the algorithm.

**Aimed sensitivity level**	**Sensitivity (Actual) (%)**	**Specificity (%)**	**Positive predictive value (%)**	**Negative predictive value (%)**	**Balanced accuracy**	**F1-score**
Sensitivity of 1	100	40.2	48.3	100.0	0.701	0.651
Sensitivity of 0.975	97.5	49.6	51.9	97.2	0.735	0.677
Sensitivity of 0.95	94.9	53.0	53.0	94.9	0.739	0.680
Sensitivity of 0.90	88.8	67.4	60.3	91.5	0.781	0.719
Sensitivity of 0.85	84.3	73.1	63.6	89.3	0.787	0.725
Sensitivity of 0.80	79.2	79.6	68.4	87.3	0.794	0.734
Sensitivity of 0.75	71.6	84.1	71.6	84.1	0.779	0.716
Best balanced accuracy	77.7	79.9	68.3	86.5	0.788	0.727

*AUC, Area Under the Receiving Operating Curve*.

All these results provide an estimate of the generalized performance of the algorithm when applied in new subjects which were not included in the sample used to develop the model and that have been evaluated in distinct recruiting sites.

On the server we employed in our study, training took around 12 h for each of the 5 test folds, with a total training time of 2 days and a half. Instead, the computational time necessary to calculate the prediction using the ensemble of machine learning algorithms is <1 s for each case in each fold.

### Importance of Predictors

**Figure 2:**
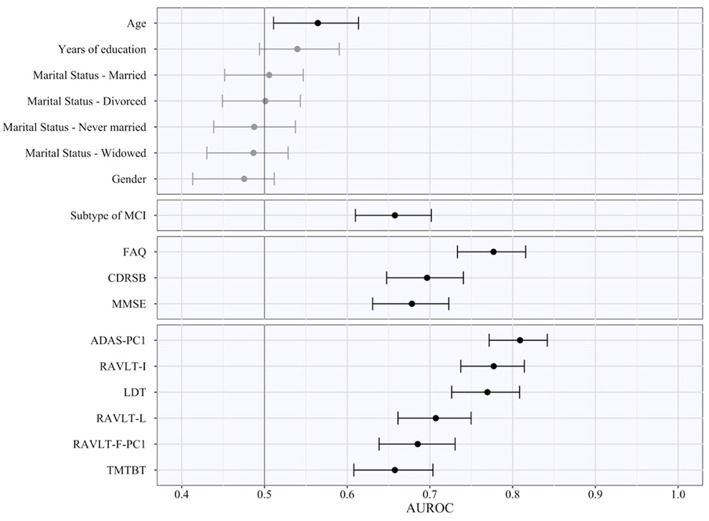
Area under the receiving operating curve of individual predictors. The figure indicates the pooled test AUROC and its 95% bootstrap CI when prediction is made considering each predictor singularly. Predictors are grouped according to conceptual domains, which in descending order are sociodemographic characteristics, subtype of MCI, clinical scale scores, and neuropsychological test scores. Non-significant AUCROC (i.e., the lower bound of the CI is lower than or equal to 0.5) are in gray, significant ones in black.

The AUROC of each of the various features obtained by pooling the results in the five test subsamples is reported in Table 6, ranked from the highest to the lowest AUROC, and in Figure 2, subdivided based on type of the features (i.e., sociodemographic, subtype of MCI, clinical, and neuropsychological tests). These represent an estimate of the generalized predictive performance achievable using each feature singularly.

**Table 6:** Individual test pooled AUROC of each feature.

	**AUROC**	**95% Boostrap CI**	
ADAS-PC1	0.809	0.772	0.842
RAVLT-I	0.777	0.737	0.814
FAQ	0.777	0.733	0.816
LDT	0.770	0.726	0.808
RAVLT-L	0.707	0.661	0.750
CDRSB	0.697	0.648	0.740
RAVLT-F-PC1	0.685	0.639	0.730
MMSE	0.678	0.631	0.723
Subtype of MCI	0.658	0.610	0.702
TMTBT	0.658	0.608	0.704
Age	0.564	0.511	0.614
Years of education	0.540	0.494	0.590
Marital status—Married	0.506	0.452	0.547
Marital status—Divorced	0.501	0.449	0.543
Marital status—Never married	0.488	0.439	0.537
Marital status—Widowed	0.487	0.430	0.529
Sex	0.475	0.413	0.512

Sociodemographic characteristics resulted the least relevant, with age being the sole with a statistically significant AUROC (lower bound of the 95% bootstrap CI higher than 0.50) even if quite small in magnitude (AUROC_age_ = 0.57). Instead, both subtypes of MCI and CDRSB demonstrated a better predictive performance (AUROC_MCI_ = 0.66; AUROC_CDRSB_ = 0.70), and FAQ a high AUROC of 0.78. Among the neuropsychological test scores, some of them also proved to have a high predictive capability even when used as individual predictors. The ADAS-PC1 achieved an AUROC of 0.81, RAVLT-I of 0.78, and LDT of 0.77. All other neuropsychological test scores resulted with an inferior AUROC (minimum AUROC: AUROC_TMTBT_ = 0.66).

Of notice, the most relevant of the predictors, e.g., ADAS-PC1, resulted having a significantly lower test AUROC than the one demonstrated by the algorithm we developed (higher bound of the 95% bootstrap CI of ADAS-PC1 = 0.84 < lower bound of the 95% bootstrap CI of the algorithm = 0.85).

## Discussion

The aim of the current study was to develop a new machine-learning algorithm to allow a 3-year prediction for conversion to AD in subjects diagnosed with MCI.

Considering an imminent necessity of being able to discriminate which MCI subjects will progress to AD from those who will not, as soon as in a few years the first effective treatments will be probably available (2), our algorithm has been designed to be used as a prognosis support tool for MCI patients, which is cost-effective and easily translatable to clinical practice. This would allow timely planning of early interventions for such individuals. Further, our algorithm can be employed as a tool during the recruitment of MCI subjects for clinical trials which aim to investigate innovative treatments of AD. The opportunity to recruit only subjects at true risk of future conversion to AD—who most likely show the earliest brain changes underlying AD pathology—will drastically reduce the costs to run such clinical trials and result in improved outcomes.

In contrast with many of the machine-learning approaches that have been previously presented, our algorithm aimed to achieve good predictive performance based only on a reduced set of sociodemographic characteristics, clinical information, and neuropsychological tests scores. It does not rely on information coming from procedures that are currently still expensive, invasive or not widespread available in many clinical settings, such as neuroimaging techniques, lumbar puncture, and genetic testing.

The algorithm was developed using a sample of MCI subjects recruited in the ADNI study and we applied a site-independent testing protocol in order to obtain results which represent a better estimate of the expected performance when the algorithm is applied in distinct clinical centers. To the best of our knowledge, this is the first algorithm that was tested ensuring independence between the train and test sets regarding the sites where the subjects were recruited from.

Even using such a rigid testing protocol, the algorithm demonstrated a high predictive performance, showing a test AUROC of 0.88, a sensitivity of 77.7%, and a specificity of 79.9% when the classification threshold was optimized to achieve the best possible balanced accuracy. Of particular interest is the achievement of 40.2%/53% specificity and 48.3%/53% positive predictive value when the threshold was further optimized to achieve a sensitivity of respectively, 100 and 95%. These results support the utility of our algorithm especially as a potential screening tool, i.e., an algorithm that can provide a marginal number of false negative predictions at the cost of a higher number of false positives. Thus, our algorithm would turn out to be particularly useful in case another more accurate, and especially more sensitive tool will become available, however which requires additional expensive or invasive-to-collect information. In such case, our algorithm can be used as a first step to significantly reduce the number of subjects which require examination using more precise, yet less easily applicable procedures at a later stage. Considering an expected conversion rate of 20–40% from MCI to AD in 3 years, the expected percentage of subjects confidently predicted as non-converters would be estimated 32–24% subsequently, leaving only the remaining 68–76% of subjects with the necessity of further investigations.

Making a proper comparison of our algorithm with all others previously published is not a trivial task, especially considering the different and reduced level of independent validation most of these algorithms have undergone so far.

In some studies, algorithms which used as predictive information some type of functional brain imaging, such as PET and fMRI, and/or CSF investigations demonstrated particularly high cross-validated performance, with AUROCs close to 0.95 (18, 19). A recent study presented an algorithm based on regional information from a single amyloid PET scan which demonstrated a test performance of an AUROC of 0.91 and an unbalanced accuracy of 0.84 in the ADNI sample for a prediction of conversion in 2 years (43), thus showing a higher predictive performance than what was achieved by our algorithm.

In addition, some studies which used only structural MRI also demonstrated high cross-validated [i.e., (18, 19): AUROC = 0.932; balanced accuracy = 0.886] and nested cross-validated performance [(44): sensitivity = 85%; specificity = 84.78%]. Similarly, high cross-validated results were found by other studies who combined structural MRI with clinical and neuropsychological information [i.e., (7, 11–19)]: AUROC = 0.902; balanced accuracy = 80.5%) In addition, a recent study (45) presented a highly performing deep learning algorithm (AUROC = 0.925; accuracy = 86%; sensitivity = 87.5%; specificity = 85%) and, to the best of our knowledge, this is the only available study using structural MRI in which a proper testing of the algorithm was performed.

Some particularly promising cross-validated results were also found in some studies which considered also APOE genotyping, together with EEG, [(46): AUROC = 0.97; sensitivity = 96.7%; specificity = 86%] or blood biomarkers (7, 11, 17): balanced accuracy = 92.5%). Thus, the use of brain imaging, CSF, and/or other biomarkers as predictive information may have, to some degree, resulted in a better predictive performance compared to our algorithm, which did not use any of these types of information.

While the results of the previous studies indicate that neuroimaging biomarkers hold great promise for predicting conversion to AD, the performance increase gained by including biomarker information is questioned and much debated (15, 47, 48). Instead, neuropsychological measures of cognitive functioning are possibly equally excellent predictors of progression to dementia. For example, in a study by Fleisher and colleagues, common cognitive tests provide better predictive accuracy than imaging measures for predicting progression to AD in subject with moderate stages of amnestic MCI (48), and in another study by Clark and colleagues, models developed using only socio-demographic information, clinical information and neuropsychological test scores (focusing on verbal fluency scores) resulted in an AUROC score of 0.87 and a balanced accuracy of 0.84, while including brain imaging did not significantly improve this performance (AUROC = 0.81, accuracy = 0.83) (15).

Moreover, the cost of the standard procedure in the clinical process of diagnosing AD (which entails the clinical consultation, including the patient's administrative admission, anamnesis, physical examination, neuropsychological testing, test evaluation and diagnosis conference & physician letter) is relatively low at an estimated 110 € (US$115) on average, while the use of additional advanced technical procedures, such as blood sampling, CT, MRI, PET & CSF procedures, which are required following deficits in neuropsychological test results and depends on the patient's suspected diagnosis of MCI, AD or other dementia types (which is increasingly associated with higher frequencies of using cost-intensive imaging & CSF procedures), drives costs up to 649 € (US$676) in case of an AD diagnosis according to a study in a German memory clinic (49).

In this regards, the use of advanced technological procedures, rather than clinical consultation and neuropsychological testing, is driving costs in the diagnostic process and as such, will also increase the costs of predictive algorithms based on information of imaging, blood sampling or CSF procedures compared to those algorithms that rely only on sociodemographic, clinical, and neuropsychological predictive information, like the one we present in this study. In addition, even if nowadays some forms of neuroimaging investigations are often routinely performed, for example in order to evaluate other potential comorbidities such as neurovascular problems or regional atrophies, and thus such information may result already available without additional costs, a clear evidence of its relevance to improve predictions based only on neuropsychological and clinical measures is still lacking, as it has already been discussed above, and still requires further investigations.

Additionally, our algorithm demonstrated similar predictive performance compared to other top-performing algorithms based only on sociodemographic, clinical, and neuropsychological predictive information. For example, in a first study by Clark and colleagues, they used only a simple cross-validation protocol to investigate the performance of their algorithm to make prediction of conversion at 1 year or more (AUROC = 0.88, balanced accuracy = 0.84) (14), while in another study they used a more sound nested cross-validation protocol to investigate the predictive performance of their algorithm at 4 years (AUROC = 0.87, balanced accuracy = 0.79) (15).

Our results originate from a proper testing protocol and represent a better unbiased estimate of the generalized performance of the algorithm. Only a very small number of machine learning algorithms for the prediction of conversion from MCI to AD were subjected to a proper testing protocol, rather than only a cross-validation protocol, which limits the soundness of the evidence of their predictive performance. As such, apart from (43, 45), all the previously mentioned results may be optimistically biased estimates of the generalized performance of such algorithms as a proper testing protocol was not applied.

We previously presented another machine learning algorithm that performs a prediction of conversion to AD in MCI subjects (20, 21). However, the algorithm described here has distinct characteristics and can be considered at a more advanced stage of validation. First, the current algorithm does not require any neuroimaging information, while our previous method relied on a clinicians' rating of the atrophy in three brain structures, evaluated by observing standardized images coming from a structural magnetic resonance. Structural magnetic resonance is widespread also in clinical settings nowadays, it is less expensive than other neuroimaging evaluation such as functional magnetic resonance and positron emission tomography, and the use of a clinician-administered visual scale allows to bypass the obstacles related to the non-automatic calibration of data coming from different magnetic resonance scanners. Nevertheless, the fact that our new algorithm does not necessitate any magnetic resonance evaluation makes its use even more easily translatable in practice, and less expensive. Moreover, even though our former algorithm showed higher cross-validated performance [AUROC = 0.91, sensitivity = 86.7% and specificity = 87.4% at the best balanced accuracy) (20)], a solid testing of its performance is still lacking and, at the moment, only a preliminary evidence via a transfer learning approach is available (21). Instead, the protocol applied in the current study provides a better and sounder evaluation of the actual predictive performance of this new algorithm.

Beyond testing the algorithm's predictive accuracy, we also aimed to provide a first indication of the importance of the variables used as predictors. The opportunity to provide an explanation of how the model works and performs its prediction is crucial to foster its application in clinical practice (10). However, given the architectural complexity of the algorithm we developed, this is not a straightforward task. Several different approaches have been proposed, all of them providing different, and only a partial explanation of an algorithm's functioning (50). Thus, we decided to leave complex and more extensive investigations to a future study which will be fully dedicated to this goal. Instead, we simply investigated the predictive role of each predictor individually, which can evidence the amount of predictive information carried by each predictor. However, it does not allow to identify potential interactions among multiple predictors that could have been modeled by the algorithm and that can relevantly contribute to its high predictive performance.

In line with the evidence in our previous study (20), sociodemographic characteristics seem not to be particularly relevant in discriminating cAD and NC MCI subjects. Furthermore, in both studies, age was the sole of these characteristics showing a significant, even if very limited, predictive power. Also, sociodemographic characteristics resulted to be the most often discarded features by the feature selection strategies we applied in our study, once again suggesting their poor predictive relevance.

Instead, the clinical scale scores, the subtype of MCI, and the neuropsychological test scores resulted markedly predictive. Their test AUROC ranged from 0.658 to 0.809, and even the least predictive of them had a 95% CI higher than 0.6. The evidence of their predictive importance was expected. These features measure core elements of the progressive decline leading to a full manifestation of AD, such as the memory and other cognitive functions deterioration, and the consequent functional impairment.

In our algorithm, as well as in several previously presented algorithms which included clinical, and neuropsychological predictors, some of these were also reassessed at later follow-ups in order to investigate when a conversion to AD occurred after the baseline assessment. As a matter of facts, MMSE and CDR scores below certain cut-offs and a cognitive impairment in at least two cognitive domains are necessary criteria to receive a diagnosis of probable AD, evidencing a conversion from MCI to AD. Using some measures at baseline to predict the same or related measures at a future follow-up time is a strategy at the foundation of time-series analyses (i.e., autoregressive models). The same measure may result correlated to itself at different future times (i.e., autocorrelation), thus making relevant predictive information at the disposal of the predictive model. Instead, in other occasions, a measure may result uncorrelated to itself across different times of assessment. The result of a significant individual predictive performance of all neuropsychological tests, MMSE, and CDR baseline scores evidences the former in our data, and it may generally be interpreted as that the more severe is the level of impairment reached by a subject, the higher becomes the probability of its progression until a conversion to AD within the following 3 years. The use of such autocorrelated information as predictors may have relevantly contributed in the high performance achieved by our as well other algorithms which included them, compared to others which did not (51, 52).

Moreover, the first principal component of the three ADAS scores, which resulted in the most individually important predictor, demonstrated a test AUROC significantly lower than the one achieved by the entire algorithm. The results of our, as well as other previous studies, had already showed that machine learning algorithms can effectively be used to combine these individual pieces of information, providing a better identification of cAD among MCI subjects than what it would be possible using each of them singularly (14, 15, 20, 21, 47).

Our study has some limitations that should be taken into account and that will be addressed in the future stages of our research. First, even if we iteratively ensured that the subjects used for testing were always recruited in different sites than those used in the development of the algorithm, it is important to note that all the ADNI recruiting sites were located in the USA or Canada. Even if this can be considered an important step forward toward the demonstration of the generalized performance of the proposed algorithm, still these sites may not be completely representative of the entire population of centers in which the algorithm may aspire to be used. Our aim was to develop an algorithm that may be applied also beyond US and Canada centers only, and perhaps also clinical centers without any research inclinations. MCI subjects referring to these extended range of centers might have peculiar characteristics and the algorithm might show reduced predictive accuracy when applied to them. In order to at least partially address this potential bias, we plan to first test and then re-optimize our algorithm using further datasets coming from the several international replications of the North American ADNI (https://www.alz.org/research/for_researchers/partnerships/wwadni). In addition, inclusion and exclusion criteria may have excluded from ADNI, and in turn from our analyses, some MCI subjects with peculiar characteristics, e.g., MCI subjects with high level of depression or currently taking some of the medications that excluded for admission to the study. Once again, the algorithm might show reduced predictive accuracy when applied to them and further testing in a less selected sample should be performed before a safe use of the algorithm can be guaranteed with these peculiar MCI subjects.

Furthermore, our final algorithm is based on an ensemble of several lower-level machine learning algorithms, including some that use the entire initial set of predictors as feature set. Thus, all predictors currently remain necessary to be assessed, even if some of them may contribute poorly or even not at all to the prediction. Although the ensembling approach we used may have effectively prevented that such irrelevant predictors decreased the algorithm accuracy, a further reduction of the amount of information necessary to be assessed and used by the algorithm would permit to reduce the costs associated with its application. At the same time, our algorithm may have missed to take into account relevant pieces of information that can improve the accuracy of its predictions.

It should be also noted that compensatory neurophysiological mechanisms, including for instance cognitive reserve factors such as bilingualism that are latent in MCI subjects, might result in misclassifications of MCI converters and non-converters (53, 54). It would be important to take this into account for predictive models, like ours, that exclusively relies on quantitative psychological test scores to predict the conversion to AD in MCI patients, as these compensatory brain mechanisms might not be reflected during neuropsychological testing and perhaps potentially impact the performance of the algorithm.

Finally, our algorithm currently operates 3-year predictions in subjects that already manifest MCI. As the new arriving treatments are expected to be the more effective the earlier they will be started, algorithms that can perform accurate predictions at even earlier stages of deterioration than MCI, and in a longer time frame, will be of particular relevance. A preliminary attempt has already been done in our previous study (20), employing also a sample of subjects with Pre-mild Cognitive Impairment (55), as well as in other previous studies which developed algorithm that aimed to make predictions for period longer than 3 years (11, 15). Future steps in our research will take into account this necessity, exploring the opportunity of making predictions at longer time periods and in earlier-stage subjects.

## Conclusions

We developed an algorithm to predict 3-year conversion to AD in MCI subjects, based on a weighted rank average ensemble of several supervised machine learning algorithms. It demonstrated high predictive accuracy when tested via a sound train/test split protocol, exhibiting especially good predictive performance when the algorithm was optimized as a screening tool. Predictions are performed using only a restricted set of sociodemographic characteristics, clinical information, and neuropsychological test scores, which makes its application of easy translation into clinical practice, as well as useful in improving the recruitment of MCI subjects at true risk of conversion to AD in clinical trials.

It is important to conclude highlighting that any prediction, including those provided by machine learning algorithms, is probabilistic in its nature and always comes with a certain degree of imprecision. The advantage of in the potential use of algorithmic decision-making tools is that such imprecision is defined by a known and objectively investigated degree of confidence. However, in order to guarantee such confidence, several and continuous tests of an algorithm have to be performed before its application can be safely recommended. Further tests and optimizations will follow this study in the attempt to provide additional evidence of its accuracy in generalized applications, and to improve its cost-effectiveness.

## Data Availability

Data used in the preparation of this article were obtained from the ADNI database (adni.loni.usc.edu), which is easily available for download from the Laboratory of Neuroimaging (LONI) website to the research public.

## Ethics Statement

The study procedures were approved by the institutional review boards of all participating centers to the Alzheimer's Disease Neuroimaging Initiative, and written informed consent was obtained from all participants or their authorized representatives.

## Author Contributions

MG contributed to the design of the work, the design and execution of the analyses, interpretation of results, drafting of the paper and knowledge communication. NR contributed to the design of the work and the analyses, interpretation of results and drafting of the paper, as well as general project management, knowledge utilization and communication, interactions with research professionals. DL contributed to the initial conception of the work and revising the manuscript. DC, KS, and GP contributed supervising the work and revising the manuscript. MD contributed to the design of the work and the analyses, interpretation of results, supervising the work, and revising the manuscript. All authors read and approved the final manuscript.

### Conflict of Interest Statement

The authors declare that the research was conducted in the absence of any commercial or financial relationships that could be construed as a potential conflict of interest.

## Footnotes

^1^Our approach based on a division in 5 site-independent test subsets and a 10-fold cross-validation applied within each of them actually mimics the popular nested cross-validation approach, which is based on the nesting of an inner (in our case, the 10-fold cross-validation) and outer cross-validation loops (in our case, the site-independent test subsets). However, even if identical in its structure, in our study we did not compare the outer loop performances obtained re-applying nested cross-validation to different competing strategies (i.e. different machine learning techniques or ensembling approaches) in order to identify the best algorithmic approach, which is the primary reason for which any type of (cross-)validation strategy is employed. Instead, we *a-priori* chose to use all of the 52 models we developed and to ensemble them with the average weighted ranks strategy. Thus, differently from what is done with nested cross-validation, the performance we observed in the outer loop was not used to take any choice about the development of the algorithm but only to provide a final estimate of the performance of our algorithm. For this reason, such final performance estimate can be safely considered a test instead of a validation of the performance of our algorithm, making the use of the term nested cross-validation not entirely appropriate and potentially misleading.

## Abbreviations

AD: Alzheimer's disease
ADNI: Alzheimer's Disease Neuroimaging Initiative
ADAS: Alzheimer's Disease Assessment Scale
ADAS11: Cognitive Subscale (11 items) Alzheimer's Disease Assessment Scale
ADAS13: Cognitive Subscale (13 items) Alzheimer's Disease Assessment Scale
ADASQ4: task 4 of the Cognitive Subscale (11 items) Alzheimer's Disease Assessment Scale
AUROC: Area Under the Receiving Operating Curve
BCa: bias-corrected and accelerated
cAD: converters to probable Alzheimer's disease
CDR: Clinical Dementia Rating Scale
CDRSB: Sum of Boxes score of the Clinical Dementia Rating Scale
CI: confidence interval
CSF: Cerebrospinal fluid
DIGIT: Digit Span Test score
EN: Elastic Net
FAQ: Functional Activities Questionnaire
GTB: Gradient Tree Boosting of Decision Trees
kNN: k-Nearest Neighbors algorithm
LDT: Logic Memory subtest of the of the Wechsler Memory Scale-Revised
LR: Logistic Regression
MLP1-Adam: Multi-Layer Perceptrons with one hidden layer and trained with adam algorithm
MLP1-Batch: Multi-Layer Perceptrons with one hidden layer and trained with full-batch gradient descent algorithm
MLP2-Adam: Multi-Layer Perceptrons with two hidden layers and trained with adam algorithm
MLP2-Batch: Multi-Layer Perceptrons with two hidden layers and trained with full-batch gradient descent algorithm
MCI: Mild cognitive impairment
MMSE: Mini-Mental State Examination
MRI: Magnetic resonance imaging
NB: Naive Bayes
NC: non-converters to Alzheimer's disease
PET: Positron emission tomography
RAVLT: Rey Auditory Verbal Learning Test
RAVLT-F: Forgetting score of the Rey Auditory Verbal Learning Test
RAVLT-I: Immediate score of the Rey Auditory Verbal Learning Test
RAVLT-L: Learning score of the Rey Auditory Verbal Learning Test
RAVLT-PF: Percent forgetting score of the Rey Auditory Verbal Learning Test
RF: Random Forest
SVM-Linear: Support Vector Machine with linear kernel
SVM-RBF: Support Vector Machine with radial basis function kernel
SVM-Poly: Support Vector Machine with polynomial kernel
TMTBT: Trail Making Test, version B.

## References

[1] Alzheimer's Disease International World Alzheimer Report 2018 The State of the Art of Dementia Research: New Frontiers. (2018).

[2] Liu JL Hlávka JP Hillestad R Mattke S Assessing the Preparedness of the US Health Care System Infrastructure for an Alzheimer's Treatment. Santa Monica, CA: The RAND Corporation (2017). Available online at: https://www.rand.org/content/dam/rand/pubs/research_reports/RR2200/RR2272/RAND_RR2272.appendix.pdf (accessed July 5, 2019). 10.7249/rhq-08-03-02 PMC6557037 31205802

[3] Alzheimer's Association 2018 Alzheimer's disease facts and figures. Alzheimers Dement. (2018) 14:367–429. 10.1016/j.jalz.2018.02.001

[4] Petersen RC Parisi JE Dickson DW Johnson KA Knopman DS Boeve BF. Neuropathologic features of amnestic mild cognitive impairment. Arch Neurol. (2006) 63:665–72. 10.1001/archneur.63.5.665 16682536

[5] Roberts RO Knopman DS Mielke MM Cha RH Pankratz VS Christianson TJH. Higher risk of progression to dementia in mild cognitive impairment cases who revert to normal. Neurology. (2014) 82:317–25. 10.1212/WNL.0000000000000055 24353333 PMC3929198

[6] Devanand DP Liu X Tabert MH Pradhaban G Cuasay K Bell K. Combining early markers strongly predicts conversion from mild cognitive impairment to Alzheimer's disease. Biol Psychiatry. (2008) 64:871–9. 10.1016/j.biopsych.2008.06.020 18723162 PMC2613777

[7] Dukart J Sambataro F Bertolino A. Accurate prediction of conversion to Alzheimer's disease using imaging, genetic, and neuropsychological biomarkers. J Alzheimers Dis. (2016) 49:1143–59. 10.3233/JAD-150570 26599054

[8] Sperling R Johnson K. Biomarkers of Alzheimer disease: current and future applications to diagnostic criteria. Continuum. (2013) 19:325–38. 10.1212/01.CON.0000429181.60095.99 23558480 PMC10563888

[9] Giannakopoulos P Alzheimer disease biomarkers: facing the complexity. J Alzheimers Dis Parkins. (2017) 7:19 10.4172/2161-0460-C1-026

[10] Perna G Grassi M Caldirola D Nemeroff CB. The revolution of personalized psychiatry: will technology make it happen sooner? Psychol Med. (2018) 48:705–13. 10.1017/S0033291717002859 28967349

[11] Agarwal S Ghanty P Pal NR. Identification of a small set of plasma signalling proteins using neural network for prediction of Alzheimer's disease. Bioinformatics. (2015) 31:2505–13. 10.1093/bioinformatics/btv173 25819077

[12] Minhas S Khanum A Riaz F Alvi A Khan SA. A Nonparametric approach for mild cognitive impairment to AD conversion prediction: results on longitudinal data. IEEE J Biomed Health Inform. (2017) 21:1403–10. 10.1109/JBHI.2016.2608998 28113683

[13] Plant C Teipel SJ Oswald A Böhm C Meindl T Mourao-Miranda J. Automated detection of brain atrophy patterns based on MRI for the prediction of Alzheimer's disease. Neuroimage. (2010) 50:162–74. 10.1016/j.neuroimage.2009.11.046 19961938 PMC2838472

[14] Clark DG Kapur P Geldmacher DS Brockington JC Harrell L DeRamus TP. Latent information in fluency lists predicts functional decline in persons at risk for Alzheimer disease. Cortex. (2014) 55:202–18. 10.1016/j.cortex.2013.12.013 24556551 PMC4039569

[15] Clark DG McLaughlin PM Woo E Hwang K Hurtz S Ramirez L. Novel verbal fluency scores and structural brain imaging for prediction of cognitive outcome in mild cognitive impairment. Alzheimers Dement. (2016) 2:113–22. 10.1016/j.dadm.2016.02.001 27239542 PMC4879664

[16] Moradi E Pepe A Gaser C Huttunen H Tohka J Alzheimer's disease neuroimaging initiative. machine learning framework for early MRI-based Alzheimer's conversion prediction in MCI subjects. Neuroimage. (2015) 104:398–412. 10.1016/j.neuroimage.2014.10.002 25312773 PMC5957071

[17] Apostolova LG Hwang KS Kohannim O Avila D Elashoff D Jack CR Jr. ApoE4 effects on automated diagnostic classifiers for mild cognitive impairment and Alzheimer's disease. Neuroimage Clin. (2014) 4:461–72. 10.1016/j.nicl.2013.12.012 24634832 PMC3952354

[18] Hojjati SH Ebrahimzadeh A Khazaee A Babajani-Feremi A Alzheimer's disease neuroimaging initiative. Predicting conversion from MCI to AD using resting-state fMRI, graph theoretical approach and SVM. J Neurosci Methods. (2017) 282:69–80. 10.1016/j.jneumeth.2017.03.006 28286064

[19] Long X Chen L Jiang C Zhang L Alzheimer's disease neuroimaging initiative. Prediction and classification of Alzheimer disease based on quantification of MRI deformation. PLoS ONE. (2017) 12:e0173372. 10.1371/journal.pone.0173372 28264071 PMC5338815

[20] Grassi M Perna G Caldirola D Schruers K Duara R Loewenstein DA. A clinically-translatable machine learning algorithm for the prediction of Alzheimer's disease conversion in individuals with mild and premild cognitive impairment. J Alzheimers Dis. (2018) 61:1555–73. 10.3233/JAD-170547 29355115 PMC6326743

[21] Grassi M Loewenstein DA Caldirola D Schruers K Duara R Perna G A clinically-translatable machine learning algorithm for the prediction of Alzheimer's disease conversion: further evidence of its accuracy via a transfer learning approach. Int Psychogeriatr. (2018) 14:1–9. 10.1017/S1041610218001618 PMC6517088 30426918

[22] Wechsler D WMS-R: Wechsler Memory Scale–Revised: Manual. San Antonio, TX: Psychological Corporation (1987).

[23] Morris JC. The Clinical Dementia Rating (CDR): current version and scoring rules. Neurology. (1993) 43:2412–4. 10.1212/WNL.43.11.2412-a 8232972

[24] Pfeffer RI Kurosaki TT Harrah CH Jr Chance JM Filos S. Measurement of functional activities in older adults in the community. J Gerontol. (1982) 37:323–9. 10.1093/geronj/37.3.323 7069156

[25] Folstein MF Folstein SE McHugh PR. “Mini-mental state”: a practical method for grading the cognitive state of patients for the clinician. J Psychiatr Res. (1975) 12:189–98. 1202204 10.1016/0022-3956(75)90026-6

[26] Rosen WG Mohs RC Davis KL. A new rating scale for Alzheimer's disease. Am J Psychiatry. (1984) 141:1356–64. 10.1176/ajp.141.11.1356 6496779

[27] Schmidt M Rey Auditory Verbal Learning Test: A Handbook. Los Angeles, CA: Western Psychological Services (1996).

[28] Wechsler D Wechsler memory scale. PsycTESTS Dataset. (1945). 10.1037/t27207-000

[29] Reitan RM Validity of the trail making test as an indicator of organic brain damage. Percept Mot Skills. (1958) 8:271–6. 10.2466/pms.1958.8.3.271

[30] Breiman L Random forests. Mach Learn. (2001) 45:5–32. 10.1023/A:1010933404324

[31] Rish I An empirical study of the naive Bayes classifier. In: IJCAI 2001 Workshop on Empirical Methods in Artificial Intelligence. New York, NY: IBM (2001). p. 41–6.

[32] Zou H Hastie T Regularization and variable selection via the elastic net. J R Stat Soc Series B Stat Methodol. (2005) 67:301–20. 10.1111/j.1467-9868.2005.00503.x

[33] Schölkopf B Smola AJ Learning with Kernels: Support Vector Machines, Regularization, Optimization, and Beyond. Cambridge: MIT Press (2002).

[34] Platt JC Probabilistic outputs for support vector machines and comparisons to regularized likelihood methods. Adv Large Margin Classifiers. (1999) 61–74. Available online at: http://citeseer.ist.psu.edu/viewdoc/summary?doi=10.1.1.41.1639

[35] Altman NS An introduction to kernel and nearest-neighbor nonparametric regression. Am Stat. (1992) 46:175–85. 10.1080/00031305.1992.10475879

[36] Kingma DP Ba J Adam. A method for stochastic optimization. arXiv [csLG]. (2014) Available online at: http://arxiv.org/abs/1412.6980 (accessed July 5, 2019).

[37] Mason L Baxter J Bartlett PL Frean MR Boosting algorithms as gradient descent. In: Solla SA Leen TK Müller K editors. Advances in Neural Information Processing Systems. Cambridge: MIT Press (2000). p. 512–18.

[38] Python Software Foundation Python Language. Available online at: http://www.python.org (accessed July 5, 2019).

[39] Pedregosa F Varoquaux G Gramfort A Michel V Thirion B Grisel O Scikit-learn: machine learning in Python. J Mach Learn Res. (2011) 12:2825–30. Available online at: http://www.jmlr.org/papers/volume12/pedregosa11a/pedregosa11a.pdf

[40] Opitz D Maclin R Popular ensemble methods: an empirical study. J Artf Intell Res. (1999) 11:169–98. 10.1613/jair.614

[41] Wolpert DH Stacked generalization. Neural Netw. (1992) 5:241–59. 10.1016/S0893-6080(05)80023-1

[42] Efron B Better bootstrap confidence intervals. J Am Stat Assoc. (1987) 82:171–85. 10.1080/01621459.1987.10478410

[43] Mathotaarachchi S Pascoal TA Shin M Benedet AL Kang MS Beaudry T. Identifying incipient dementia individuals using machine learning and amyloid imaging. Neurobiol Aging. (2017) 59:80–90. 10.1016/j.neurobiolaging.2017.06.027 28756942

[44] Guo S Lai C Wu C Cen G The Alzheimer's Disease Neuroimaging Initiative Conversion discriminative analysis on mild cognitive impairment using multiple cortical features from MR images. Front Aging Neurosci. (2017) 9:146 10.3389/fnagi.2017.00146 28572766 PMC5435825

[45] Spasov S Passamonti L Duggento A Liò P Toschi N. A parameter-efficient deep learning approach to predict conversion from mild cognitive impairment to Alzheimer's disease. Neuroimage. (2019) 189:276–87. 10.1016/j.neuroimage.2019.01.031 30654174

[46] Vecchio F Miraglia F Iberite F Lacidogna G Guglielmi V Marra C. Sustainable method for Alzheimer dementia prediction in mild cognitive impairment: electroencephalographic connectivity and graph theory combined with apolipoprotein E. Ann Neurol. (2018) 84:302–14. 10.1002/ana.25289 30014515

[47] Johnson P Vandewater L Wilson W Maruff P Savage G Graham P. Genetic algorithm with logistic regression for prediction of progression to Alzheimer's disease. BMC Bioinformatics. (2014) 15:S11. 10.1186/1471-2105-15-S16-S11 25521394 PMC4290638

[48] Fleisher AS Sun S Taylor C Ward CP Gamst AC Petersen RC. Volumetric MRI vs clinical predictors of Alzheimer disease in mild cognitive impairment. Neurology. (2008) 70:191–9. 10.1212/01.wnl.0000287091.57376.65 18195264

[49] Michalowsky B Flessa S Hertel J Goetz O Hoffmann W Teipel S. Cost of diagnosing dementia in a German memory clinic. Alzheimers Res Ther. (2017) 9:65. 10.1186/s13195-017-0290-6 28830516 PMC5568303

[50] Du M Liu N Hu X Techniques for interpretable machine learning. arXiv [csLG]. (2018) Available online at: http://arxiv.org/abs/1808.00033 (accessed July 5, 2019).

[51] Battista P Salvatore C Castiglioni I. Optimizing neuropsychological assessments for cognitive, behavioral, and functional impairment classification: a machine learning study. Behav Neurol. (2017) 2017:1850909. 10.1155/2017/1850909 28255200 PMC5307249

[52] Chapman RM McCrary JW Gardner MN Sandoval TC Guillily MD Reilly LA. Brain ERP components predict which individuals progress to Alzheimer's disease and which do not. Neurobiol Aging. (2011) 32:1742–55. 10.1016/j.neurobiolaging.2009.11.010 20005599 PMC2902777

[53] Lojo-Seoane C Facal D Guàrdia-Olmos J Pereiro AX Juncos-Rabadán O. Effects of cognitive reserve on cognitive performance in a follow-up study in older adults with subjective cognitive complaints. The role of working memory. Front Aging Neurosci. (2018) 10:189. 10.3389/fnagi.2018.00189 29997497 PMC6028562

[54] Alladi S Bak TH Duggirala V Surampudi B Shailaja M Shukla AK. Bilingualism delays age at onset of dementia, independent of education and immigration status. Neurology. (2013) 81:1938–44. 10.1212/01.wnl.0000436620.33155.a4 24198291

[55] Chao LL Mueller SG Buckley ST Peek K Raptentsetseng S Elman J Evidence of neurodegeneration in brains of older adults who do not yet fulfill MCI criteria. Neurobiol Aging. (2010) 31:368–77. 10.1016/j.neurobiolaging.2008.05.004 18550226 PMC2814904

